# Gearbox Failure Diagnosis Using a Multisensor Data-Fusion Machine-Learning-Based Approach

**DOI:** 10.3390/e23060697

**Published:** 2021-05-31

**Authors:** Houssem Habbouche, Tarak Benkedjouh, Yassine Amirat, Mohamed Benbouzid

**Affiliations:** 1 Mechanical Structures Laboratory, Ecole Militaire Polytechnique, Algiers 16046, Algeria; Houssem.habbouche@emp.mdn.dz (H.H.); tarak.benkedjouh@emp.mdn.dz (T.B.); 2 L@bIsen, ISEN Yncrea Ouest, 29200 Brest, France; 3 Institut de Recherche Dupuy de Lôme (UMR CNRS 6027), University of Brest, 29238 Brest, France; mohamed.benbouzid@univ-brest.fr; 4 Logistics Engineering College, Shanghai Maritime University, Shanghai 201306, China

**Keywords:** diagnosis, gearbox failure, linear predictive coefficients, long short-term memory, mel-frequency cepstral coefficients, convolutional neural network, sensor data fusion

## Abstract

Failure detection and diagnosis are of crucial importance for the reliable and safe operation of industrial equipment and systems, while gearbox failures are one of the main factors leading to long-term downtime. Condition-based maintenance addresses this issue using several expert systems for early failure diagnosis to avoid unplanned shutdowns. In this context, this paper provides a comparative study of two machine-learning-based approaches for gearbox failure diagnosis. The first uses linear predictive coefficients for signal processing and long short-term memory for learning, while the second is based on mel-frequency cepstral coefficients for signal processing, a convolutional neural network for feature extraction, and long short-term memory for classification. This comparative study proposes an improved predictive method using the early fusion technique of multisource sensing data. Using an experimental dataset, the proposals were tested, and their effectiveness was evaluated considering predictions based on statistical metrics.

## 1. Introduction

In recent years, the industry has undergone significant development requiring the use of increasingly complex rotating machinery [1] that needs to be monitored and maintained to avoid unplanned shutdowns [2]. Condition-based maintenance (CBM) is, therefore, the tool of choice for monitoring rotating machines’ state of health [3]. In this context, a CBM strategy includes failure detection, diagnosis, and prognosis to estimate the remaining useful life [4].

Rotating-machine diagnosis can be carried out using model- or data-based approaches [5]. While model-based techniques require the use of accurate models including machine parameters, signal-based approaches have the advantage to be driven by data without any prior knowledge about the monitored system. Signals reflecting the rotating machine’s state of health need to be acquired, however [6]. Acquired signals are further processed to extract useful information from noisy signals, which are used for failure detection and diagnosis [7].

Signal processing is ensured using different types of approaches, such as time-, frequency-, and time–frequency-domain analyses [8,9]. In this context, time–frequency is considered to be the analytical approach of choice, particularly for nonstationary signals, because of its ability to simultaneously capture features from the time and frequency domains [10]. In this field, signal-processing techniques are often combined with artificial-intelligence tools [11], to automate the diagnostic process and minimize human involvement [12,13]. In terms of artificial intelligence, machine learning is the solution of choice to effectively address major issues faced by data-driven failure detection and diagnosis approaches [14]. In this machine-learning context, convolutional neural networks (CNNs) are well-adapted for feature extraction [2], while recurrent neural networks (RNNs), with their new long short-term memory (LSTM) variant, are better suited for learning and classifying time series [15,16].

Artificial-intelligence-based diagnosis methodologies are a major focus for Industry 4.0 when based on the concept of the Internet of Things (IoT) [17], which allows for connecting everything to the Internet, such as machines and sensors [18]. To follow new Industry 4.0 trends, it is therefore necessary to consider designing autonomous expert systems [19], while benefiting from advantages of multisource data sensing for machine monitoring [20].

Several relevant proposals were recently published to design increasingly reliable monitoring systems [21] thanks to new signal-processing techniques such as linear prediction coefficients (LPC), mel-frequency cepstral coefficients (MFCC), and machine learning. Meiying et al. [16] proposed to combine a CNN and LSTM for health assessment and failure diagnosis. Features are extracted from the time and the frequency domain using the original signal and the short-time Fourier transform of the original signal, respectively. This approach was tested on two experimental datasets with a state-of-the-art comparison. In [22], Abdul et al. combined gamma tone cepstral coefficients and MFCC for feature extraction, and LSTM for gearbox failure diagnosis. However, combining two feature-extraction techniques, as shown in [16] and [22], can improve learning quality but at the price of considerably increasing prediction time, thereby limiting its real-time applications. Lei et al. [23] carried out failure diagnosis using LSTM without any prior signal processing. Acquired signals were directly linked to the LSTM input layer for feature extraction and classification. In this study, comparisons are carried out with other networks, such as multilayer perceptron (MLP), deep convolutional neural networks with wide first-layer kernels (WDCNN), and RNN. Signal data fusion was also considered. Yang et al. in [24] compared neural networks (nonlinear autoregression neural networks, NARNN, RNN, LSTM, and cross-LSTM) failure-detection performance. Failure diagnosis was performed using a sliding-window-technique-based LSTM. For rolling-bearing failure monitoring, Hao et al. [25] proposed a multisensor diagnostic framework using 1D-CNN-LSTM, 1D-CNN for feature extraction and LSTM for classification. The effectiveness of this approach was compared to that of support vector machines (SVMs), k-nearest neighbors (KNN), backpropagation neural networks (BPNNs), and CNNs. In [26], Park et al. proposed to combine two machine-learning techniques, namely, autoencoder for failure detection and LSTM for diagnosis. Combining CNN and LSTM to benefit from both advantages was proposed by An et al. [27]. This combination was effective in predicting the remaining useful life of cutting tools.

Removing the signal-processing step and extracting features by learning are common. This is, however, not always obvious according to the monitored system and corresponding failures. This is particularly the case in noisy environments, as in gearboxes where signals are too noisy, and separation becomes very difficult [28]. Indeed, as shown in [23,24], it could be more efficient to add convolution layers to benefit from their ability to extract useful information for diagnosis [25,27] or filter the signals through an autoencoder network [26], but signal processing remain the step of choice for improving the accuracy of the failure-detection process. For fault detection and isolation, Ugochukwu et al. [29] proposed to extract useful features using MFCC. Most discriminant features were then chosen as input for the classification using SVM. MFCC and LPC are widely used techniques, mainly for acoustic-signal processing. Aankit et al. [30] proposed to merge features from MFCC and LPC for spoken-language recognition. The obtained features were then used for classification using SVM, MLP, naïve Bayes, and random forest.

According to the above-discussed literature review, this paper addresses the issue of gearbox failure diagnosis, and its main contributions are the following:

- comparative study between two methodologies for gearbox diagnosis based on LPC-LSTM and MFCC-CNN-LSTM. This study highlights key features of technique suitability in an industrial context, particularly Industry 4.0;
- the use of multisensor data fusion (early fusion) to improve diagnostic reliability of the above-considered methodologies. In this context, the proposed early fusion-based fault diagnosis methodology clearly decreases training time and the data amount for storage, and improves accuracy.

The proposed methodologies were tested using a dataset collected from a specifically developed test rig, and evaluated by diagnostic metrics to highlight their industrial application interest.

This paper is organized as follows. Section 2 presents the theoretical background of the proposed methodologies. Section 3 evaluates the methodologies on the basis of an experimental dataset. A conclusion and future prospects end the paper.

## 2. Proposed Failure-Diagnosis Methodologies

**Figure 1:**
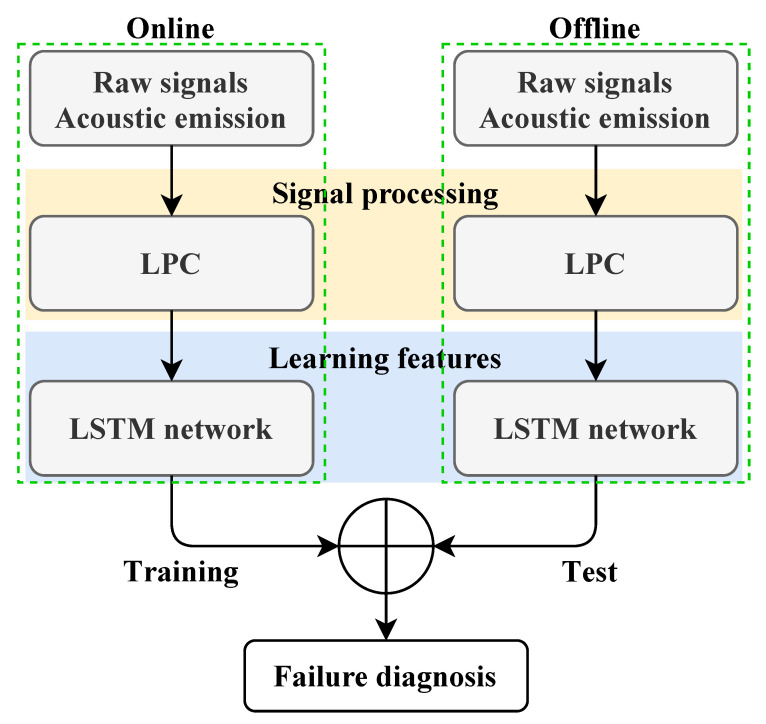
LPC–LSTM failure diagnosis methodology flowchart.

**Figure 2:**
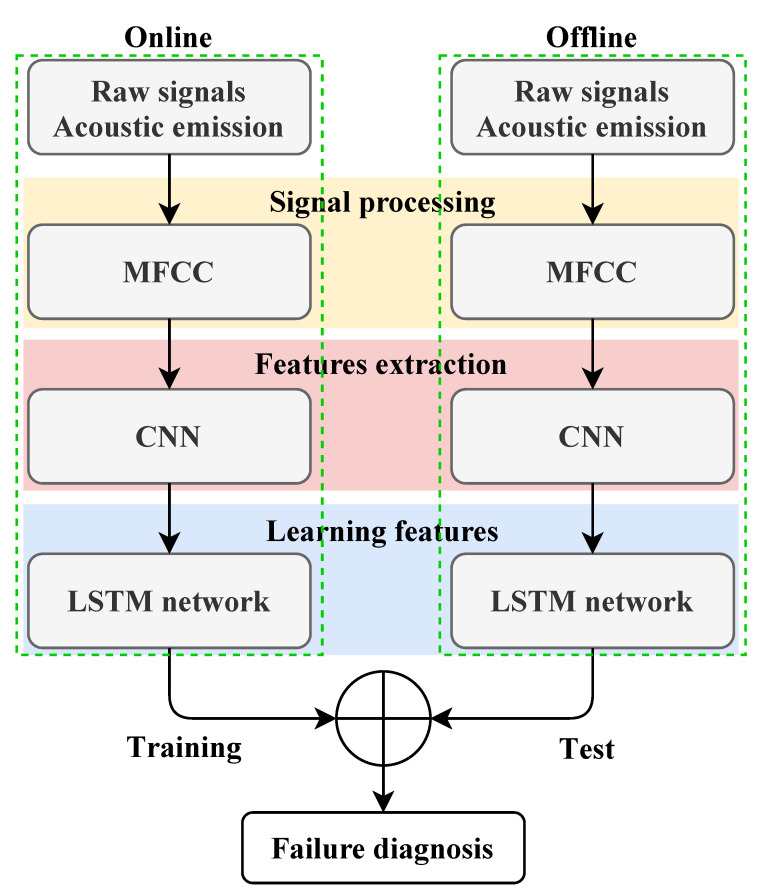
MFCC–CNN–LSTM failure diagnosis methodology flowchart.

The proposed methodologies’ flowcharts are given in Figure 1 and Figure 2, highlighting their design as expert systems for online failure diagnosis. In particular, these flowcharts illustrate the signal-acquisition step that requires sensor choice (e.g., accelerometer and microphone), handling the sensor position issue, and considering their key features (sensitivity, frequency, range, etc.) and acquisition-card choice (sampling frequency, input-channel number, etc.) [31].

### 2.1. Linear Prediction Coefficients

As measured rotating-machinery signals are often nonstationary and can be highly noisy, there is a clear need for increasingly efficient signal-processing techniques to improve failure-diagnosis accuracy [32,33]. In this context, LPC, widely used especially in speech recognition for signal analysis and feature extraction, is an interesting option for investigating failure diagnosis signal processing.

LPC is based on the fact that each sample S(n) can be written as a sum of *P* past-element s(n−k), weighted with model parameters $a_{k}$ and added to a residual term Gu(n), as follows [34,35]:

(1) $$S\left(n\right)\approx a_{1}s\left(n-1\right)+a_{1}s\left(n-1\right)+...+a_{p}s\left(n-p\right);\;$$

otherwise,

(2) $$S\left(n\right)=\sum_{k=1}^{P}a_{k}s\left(n-k\right)+Gu\left(n\right)$$

Equation (2) can be reformulated into the frequency domain into a digital filter:

(3) $$H\left(z\right)=\frac{S(z)}{U(z)}=\frac{G}{1-\sum_{k=1}^{P}a_{k}z^{-k}}\;$$

Estimating S(n) can be performed by a linear approximation of the previous *p* samples:

(4) $$\hat{S}=\sum_{k=1}^{P}a_{k}s\left(n-k\right)\;$$

Prediction-coefficient determination is based on minimizing the error between the original and approximated signals:

(5) $$e\left(n\right)=S\left(n\right)-\hat{S}\left(n\right)=S\left(n\right)-\sum_{k=1}^{P}a_{k}s\left(n-k\right)$$

Obtained coefficients $a_{k}$ are the image of the processed signal that carries discriminating information among different classes. These coefficients are the inputs of the learning network.

### 2.2. Mel-Frequency Cepstral Coefficients

**Figure 3:**
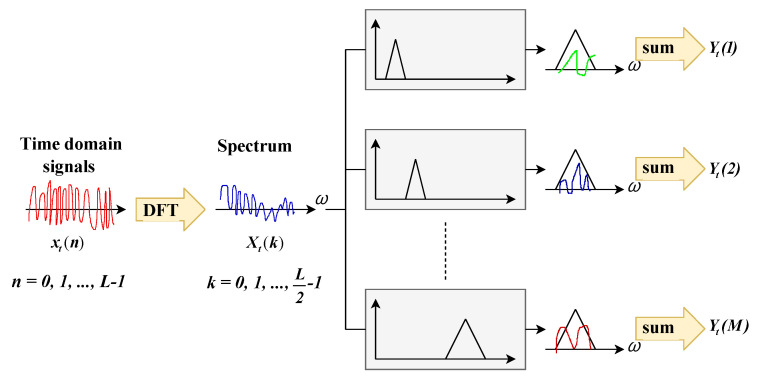
Mel-frequency cepstral coefficients.

This signal-processing technique first consists of windowing signal into samples to be as close as possible to a stationary signal. Each sample is then processed by discrete Fourier transform (DFT). Signals are then filtered to extract each level’s information. The mel-frequency spectrum uses triangular windowing that allows for calculating the energy logarithm in each filter, as shown in Figure 3. Applying a discrete cosine transform on mel-log-power allows for lastly calculating the cepstral coefficients [22].

### 2.3. Convolutional Neural Network

While several algorithms are used for feature extraction, CNNs are effective in many application domains ranging from medicine to object detection. CNNs are primarily composed of a succession of convolutional layers using different filter sizes to generate features and pooling (max and average) layers using a nonlinear downsampler to extract local features [36].

In this work, a 2D-CNN is proposed for feature extraction from MFCC spectral images to distinguish between different gearbox failures.

### 2.4. Long Short-Term Memory

As CNNs are generally unable to learn features from nonstationary signals such as vibratory measurements, RNNs were introduced [16]. They, however, suffer from gradient vanishing at the training end. To tackle this issue, LSTM RNNs are the new variant.This allows for controlling the generated information flow, and solves the gradient-vanishing issue with dynamic learning features [13].

LSTM gate equations are formulated as follows [16].

Input gate:

(6) $$i_{t}=\sigma \left(W_{ix}x_{t}+W_{ih}h_{t-1}+b_{i}\right)$$

(7) $${\tilde{C}}_{t}=\tanh\left(W_{cx}x_{t}+W_{ch}h_{t-1}+b_{c}\right)$$

Forgetting gate:

(8) $$f_{t}=\sigma \left(W_{fx}x_{t}+W_{fh}h_{t-1}+b_{f}\right)$$

Output gate:

(9) $$C_{t}=i_{t}\times {\tilde{C}}_{t}+f_{t}\times C_{t-1}$$

(10) $$o_{t}=\sigma \left(W_{ox}x_{t}+W_{oh}h_{t-1}+b_{o}\right)$$

**Figure 4:**
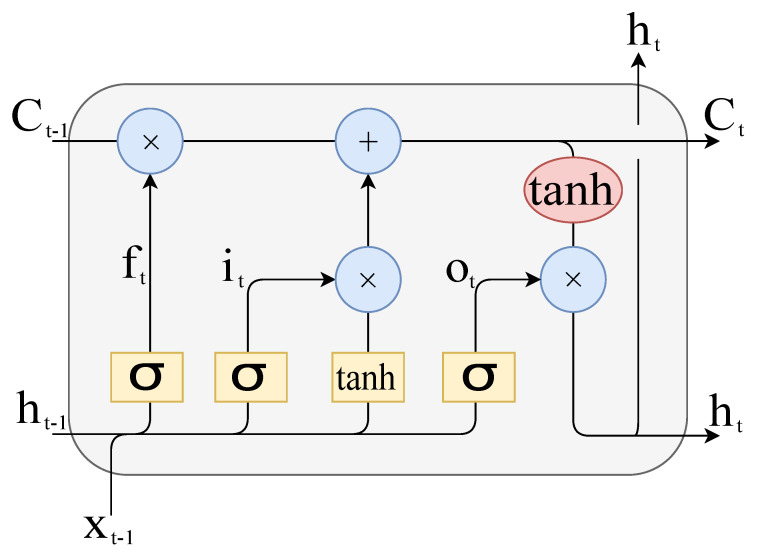
Long short-term memory cell.

Next LSTM state:

(11) $$h_{t}=o_{t}\times \tanh\left(C_{t}\right),$$

where σ and tanh are the sigmoid and hyperbolic tangent activation functions, respectively. Matrices $W_{ix},W_{cx},W_{fx},W_{ox}\in R^{N\times M}$, $W_{ih},W_{ch},W_{fh},W_{oh}\in R^{N\times N}$, and vectors $b_{i},b_{c},b_{f},b_{o},\in R^{N}$ are the (input, recurrent, and bias) learnable (input, update, forget, and output) weights, respectively, where *N* denote the size of the hidden layer per LSTM cell, and *M* is the feature size. $x_{t}$ is the current input, $h_{t-1}$ and $h_{t}$ are the previous and actual hidden state, and $C_{t-1}$ and $C_{t}$ are the previous and actual memory cell value. Equations (6) to (11) manage the flow of information in an LSTM node (Figure 4).

### 2.5. Evaluation and Classification

The proposed methodologies’ last step is failure diagnosis based on the above-defined networks. Classifications are assessed using two criteria, accuracy and confusion matrix [37], where accuracy is used for a general evaluation, and the confusion matrix is used for the detailed evaluation of each fault.

## 3. Experimental-Dataset-Based Evaluation and Validation

### 3.1. Experimental Test Bench and Dataset

**Figure 5:**
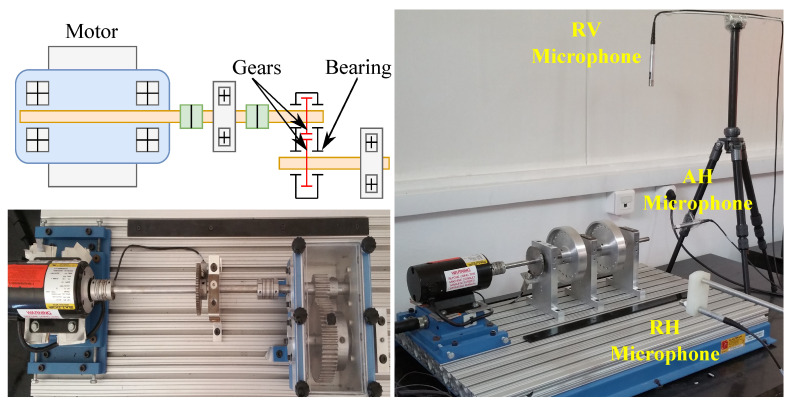
Experimental test bench.

For validation purposes, a specific test bench, namely, HTM90, including gearbox and bearing failures, was used (Figure 5). This is dedicated to the emulation of mechanical faults in rotating machines (gear, rolling, misalignment, etc.). It mainly consists of a motor, gearbox, and various healthy and faulty components to carry out fault-detection and -diagnosis tests. To build the dataset, signals were acquired through three prepolarized piezoelectric 4188-C-001 microphones from Bruël and Kjær (radial-vertical (RV), axial-horizontal (AH), and radial-horizontal (RH)). Another channel was devoted to a tachometer. The electrical signal of the microphones was acquired using a Bruël and Kjær 3050-A-060 acquisition board, which has 6 LEMO7-pin channels and a maximal sampling frequency of 50 kHz.

The testing procedure consisted of the following steps: (1) three microphones were connected to the acquisition board an using 7-pin connector cable (AO-0414); (2) the microphones’ technical characteristic specification (sensor type, sensitivity, etc.) was used in the Bruël and Kjær Pulse Labshop software; (3) lastly, acquisition frequency was set to 25.6 kHz. The main bench components and specifications were: (1) DC motor (Baldor AP7422, type 2424P, 0.25HP, 3450 rpm), and (2) speed was set to 1500 rpm (25 Hz) thanks to a tachometer connected to a digital display (speed control). The motor was connected to a drive shaft supported by a rolling platform by flexible coupling, and similarly on the other side of the shaft connected to the gearbox. This gearbox consisted of a single gear stage supported by four bearings, as shown in Figure 5.

**Figure 6:**
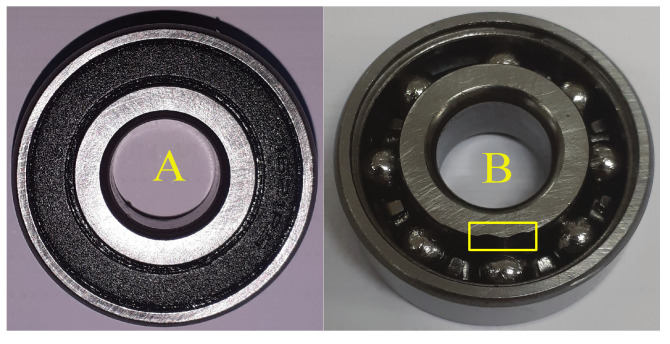
(**A**) Healthy bearing; (**B**) faulty bearing.

**Figure 7:**
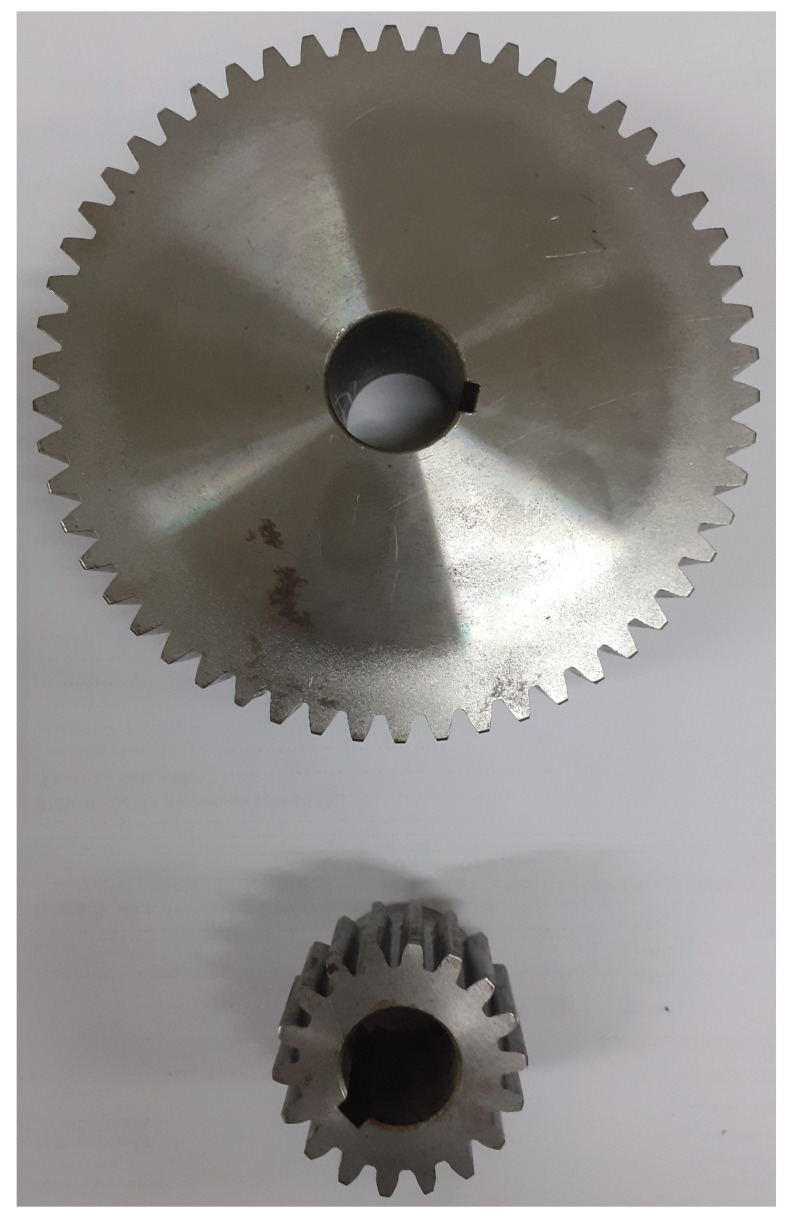
Used healthy gears.

**Figure 8:**
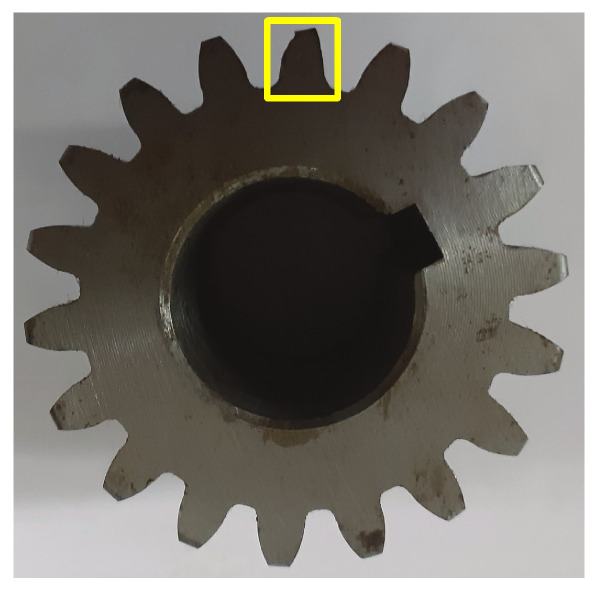
Faulty gear.

Tests were performed at room temperature (25 °C) with lubrication after each installation. The used bearings had the following specifications: 1621-RS, 12.7 mm inner diameter, 34.925 mm outer diameter, and 11.112 mm width. Healthy and faulty (inner race failure) bearings are illustrated by Figure 6. The used spur gears were Boston Gear YD54A (20° pressure angle, 54 teeth) and YD18-3/4 (20° pressure angle, 18 teeth) for gearbox input and output, respectively, as shown in Figure 7 (healthy gear); Figure 8 shows the used faulty gear.

Recording began after microphone installation over a 500 mm radius of the gearbox for each configuration shown in Table 1, on the three directions, namely, RV, AH, and RH.

A 40 s recording was adopted for each failure; each recording was split into 0.5 s pieces leading to a total of 80 samples for each failure. The test bench allowed for emulating 12 failures by combining four gear states (healthy, broken side, broken tooth, and notched) with three bearing states (healthy, inner race failure, and rusty bearing), as shown in Table 1.

**Figure 9:**
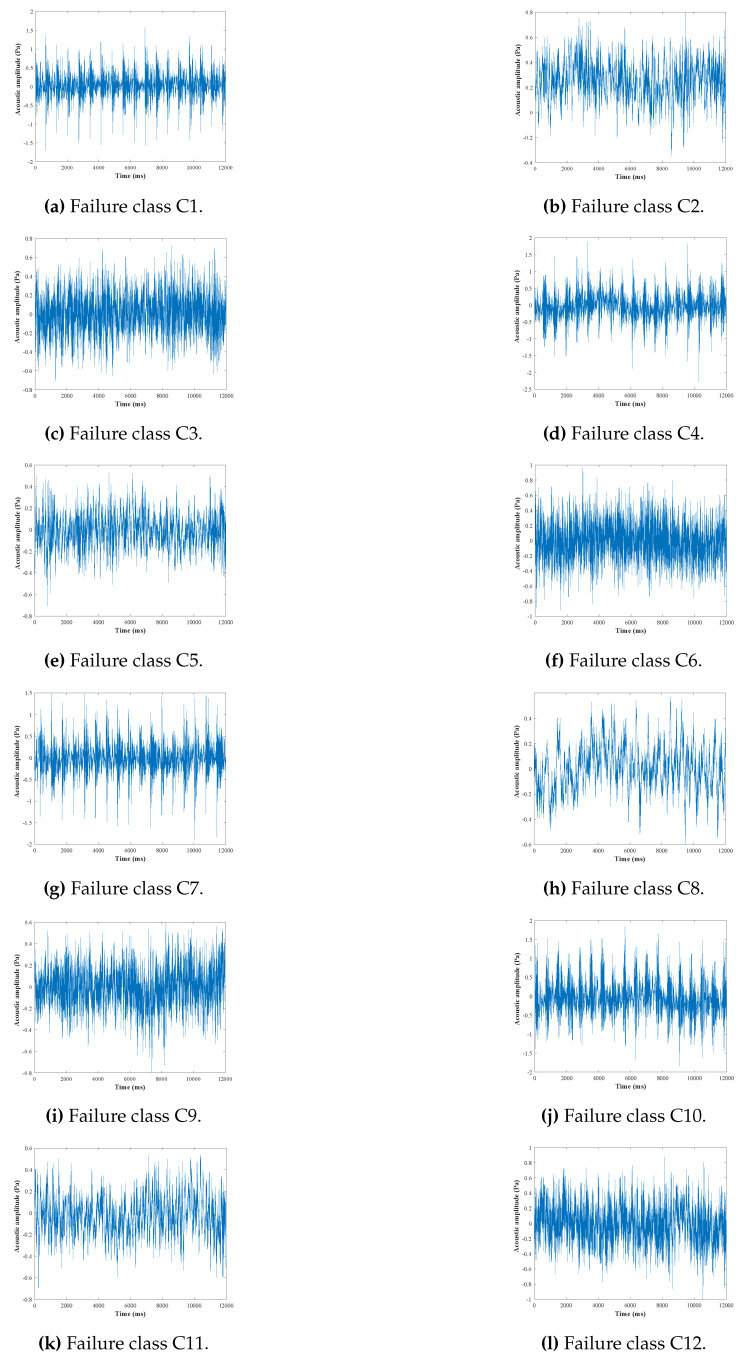
Samples of obtained signals from each failure class.

Samples of obtained signals from each failure class simulation are shown in Figure 9. These signals were later processed using MATLAB (from Matworks, licenced to Ecole Militaire Polytechnique, Algiers, Algeria).

This framework is acoustical fault diagnosis, which has several advantages over other monitoring techniques, such as vibration and current. Among these advantages are the following: (1) noncontact measuring, which can be useful in harsh and severe environments (e.g., high temperatures and corrosion) [38,39]; (2) cheap and practical technique to deploy compared to vibration- or current-based monitoring [39,40]; (3) machine diagnosis is often preceded by fault-source location by a microphone array. It is then easier to use a few microphones for diagnostic purposes [41].

### 3.2. LPC–LSTM-Based Failure-Diagnosis Methodology

**Figure 10:**
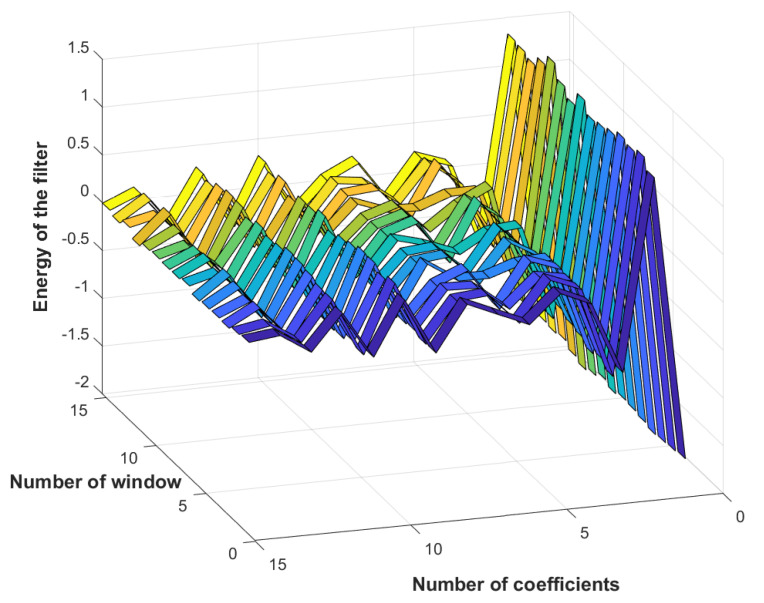
Linear predictive coefficients of first-class samples.

All the above-mentioned samples were processed by LPC to estimate the first 15 signal coefficients for the 12 considered failures, as shown in Figure 10. Afterwards, the obtained coefficients fed the LSTM network for learning. This step allowed for identifying common features between samples of the same class and feature-discriminating classes.

**Figure 11:**
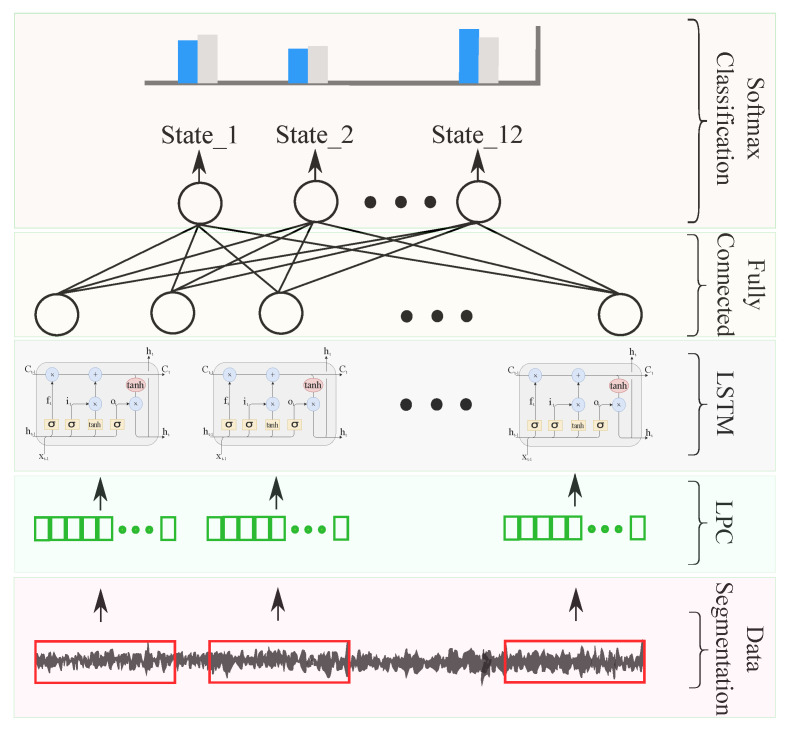
Proposed LSTM network architecture.

LSTM failure learning and classification are illustrated by Figure 11. The considered network consisted of four layers: the first for input data, a 100-node LSTM layer, a 10-node fully connected layer, and a softmax layer for classification. Regarding training, the used options were: max epochs, 100; minibatch size, 27; and initial learning rate of 0.001 with a drop factor of 0.6 every 30 epochs with the Adam solver.

### 3.3. LPC–LSTM Methodology Results and Evaluation

**Figure 12:**
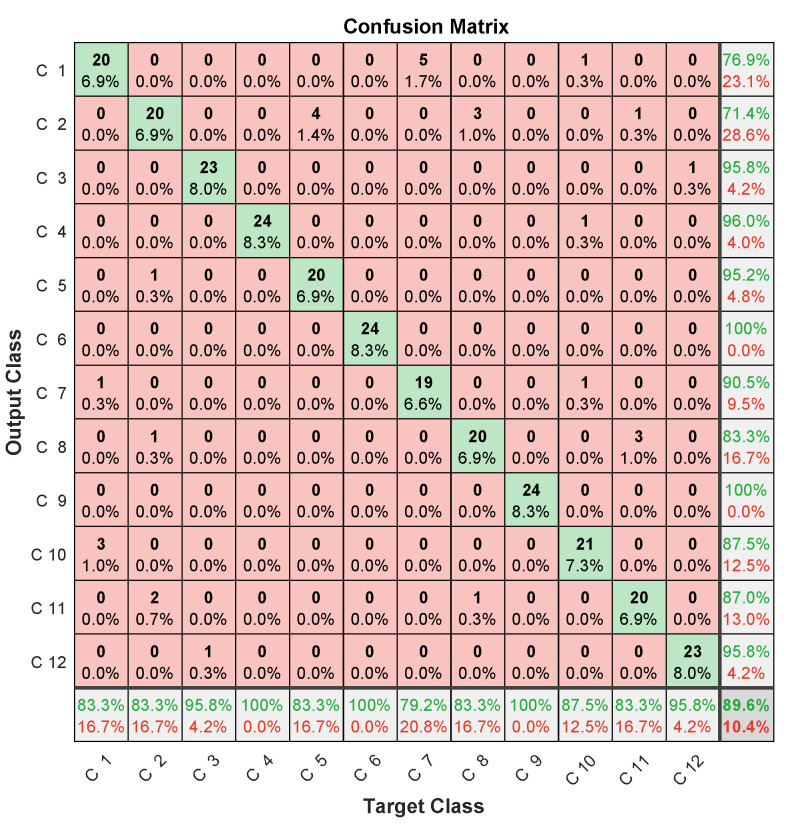
LPC–LSTM methodology confusion matrix (1st microphone).

**Figure 13:**
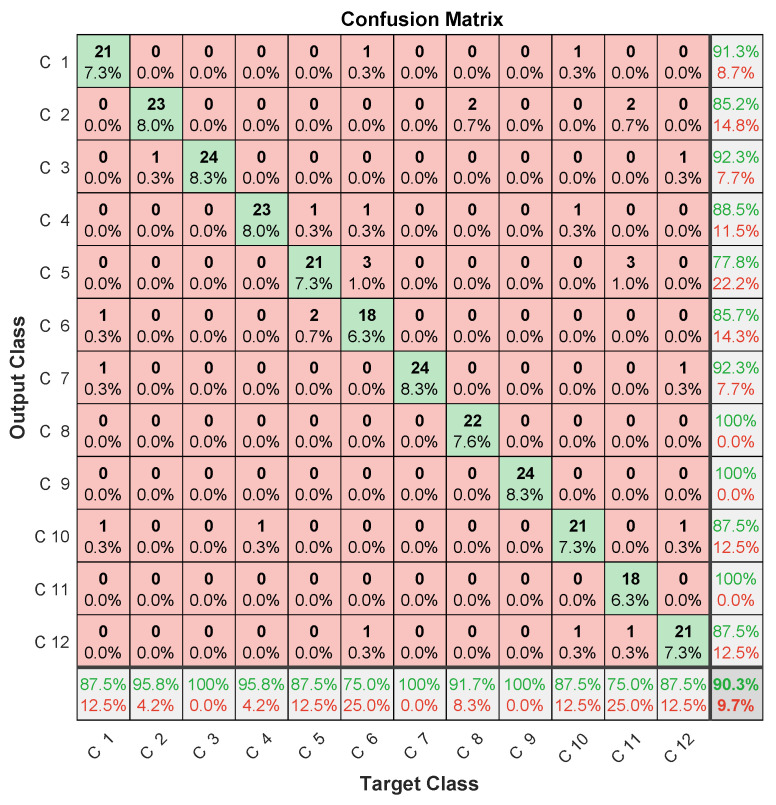
LPC–LSTM methodology confusion matrix (2nd microphone).

**Figure 14:**
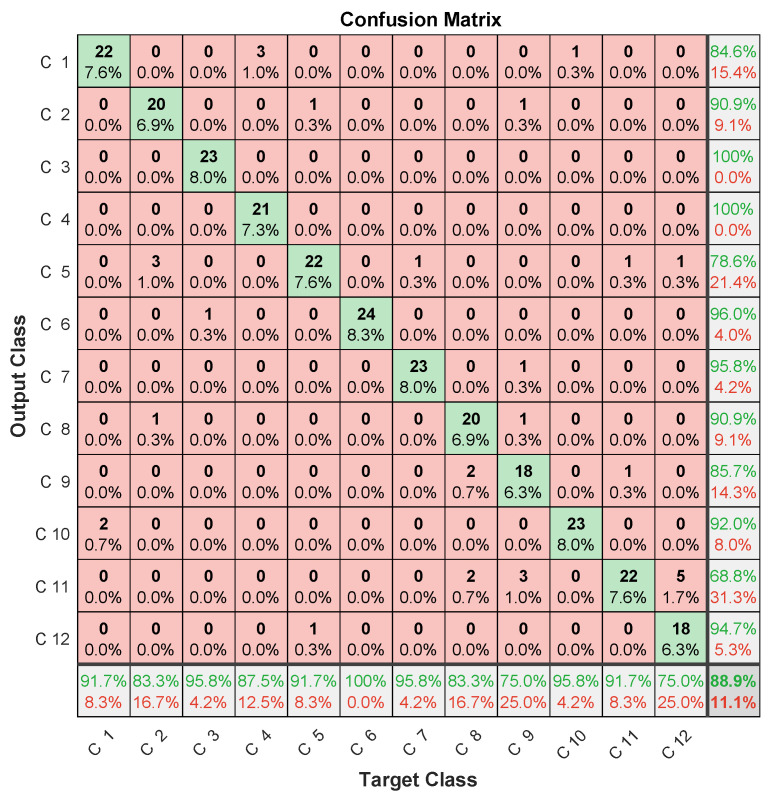
LPC–LSTM methodology confusion matrix (3rd microphone).

Specific data issued for the experimental dataset were used for testing. In this case, the three microphones’ prediction assessments are illustrated in Figure 12, Figure 13 and Figure 14 in terms of confusion matrix, and in Table 2 in terms of accuracy.

The achieved results showed quite interesting performance, with around 90% accuracy. When analyzing the confusion matrices, two misclassification types were found. The first concerned misclassified classes in one microphone, but perfectly classified in the two others. The case of the 6th failure that was perfectly classified in the first and third microphones, and misclassified 6/24 samples in the second microphone. The same applied to the 9th failure, giving 24/24 for the first and second microphones, and missing 6/24 samples for the third microphone. This led to the important conclusion that misclassifications by one microphone can be perfectly retrieved by the others.

The second misclassification type concerned failed samples in each class. For example, in the 8th class, there were 4/24 failed samples in the first microphone, of which 3/24 were in the 2nd class, while 1/24 in the 11th class. On the other hand, the third microphone failed 4/24, of which 2/24 were in the 9th class, while 2/24 others were in the 11th class. Another example concerned the 12th class, where the second microphone missed 1/24 in the 3rd class, 1/24 in the 7th class, and 1/24 in the 2nd class. On the other hand, the third microphone missed 1/24 in the 5th class, and 5/24 in the 11th class. This second type of misclassification allowed for us to highlight that samples missed in a microphone are not necessarily those missed in another.

These two types of analysis allow for concluding that classification performance could be improved by merging data from different microphones.

### 3.4. MFCC–CNN–LSTM-Based Failure-Diagnosis Methodology

MFCC is proposed for investigation, as it is specifically efficient for processing acoustic signals, which was the case of the used gearbox-failure dataset.

**Figure 15:**
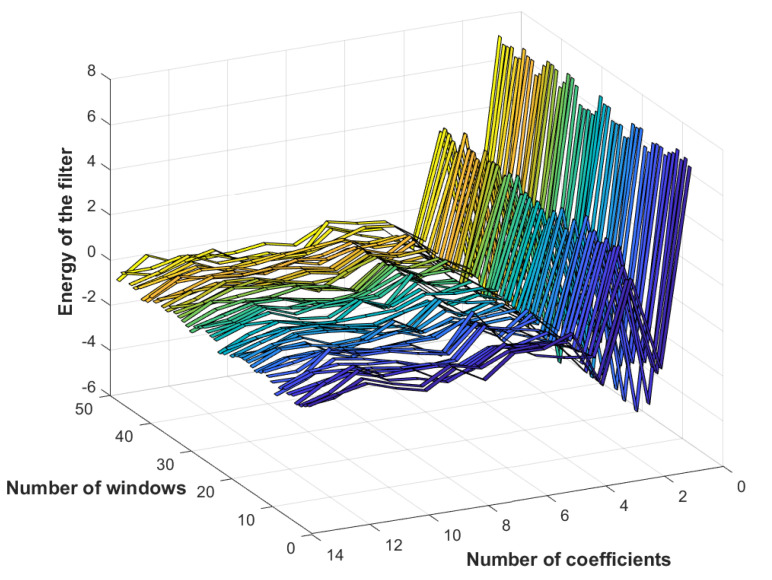
MFCC of samples from first microphone.

**Figure 16:**
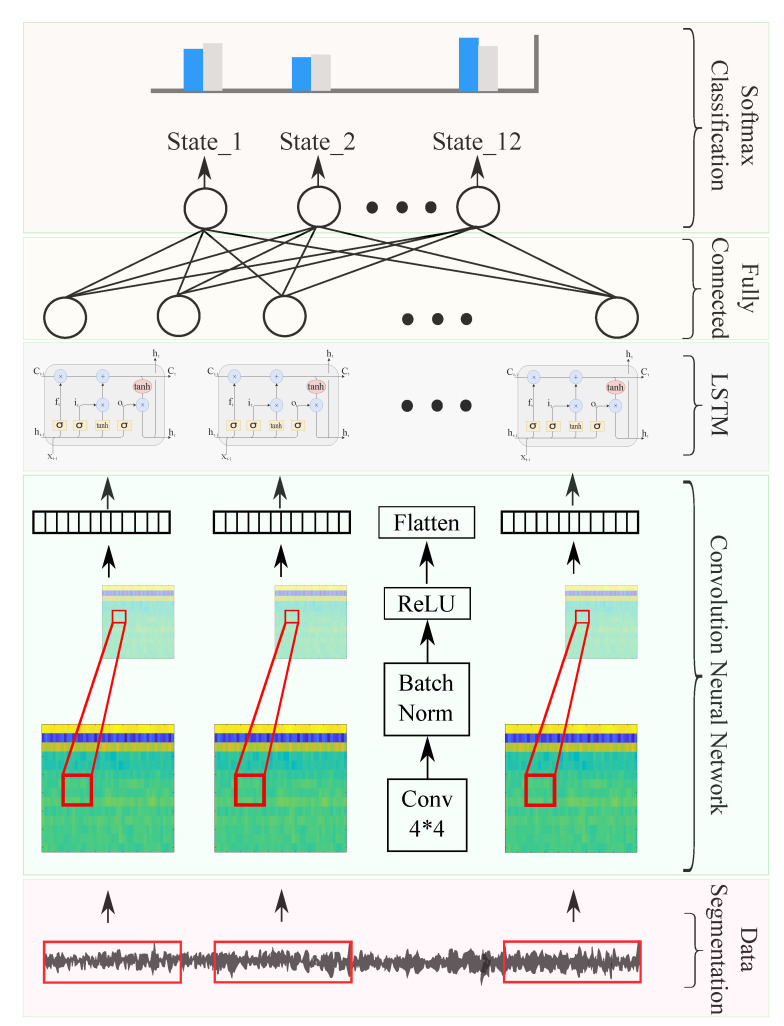
CNN–LSTM network architecture.

In this context, with a sampling frequency of 25.6 kHz, MFCC 2D spectral image outputs, illustrated in Figure 15, were used as CNN inputs for feature extraction. The used convolutional network consisted of a succession of layers, as shown in Figure 16, with a 14×48 sized 2D input layer, and a convolutional layer with stride and padding equal to 2 and 1, respectively. To enhance learning, a batch-standardization layer was used to ensure that the characteristics are in the same range. A ReLU layer was then used to cancel values below zero and obtain an output between 0 and 1. Before learning began, a flattened layer was used to align the resulting image in vector form. On this level, a specific architecture is proposed to enhance failure-diagnosis results. Convolutional operations of the above-mentioned step results are proposed. The proposed network architecture consisted of 3 layers: LSTM with 10 nodes superimposed on a fully connected layer of 12 nodes, and a softmax layer. Regarding training, the used options were an Adam optimizer, learning rate of 0.001, and minibatch size set at 27, computed on a CPU with a learning-rate drop factor of 0.6 every 30 epochs.

### 3.5. MFCC–CNN–LSTM Methodology Results and Evaluation

**Figure 17:**
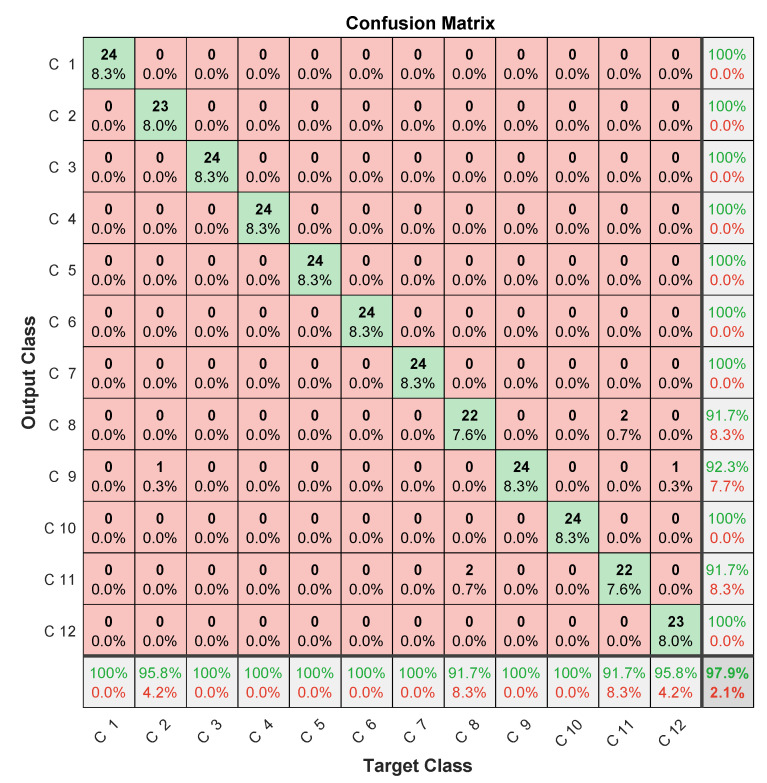
Confusion matrix (1st microphone).

**Figure 18:**
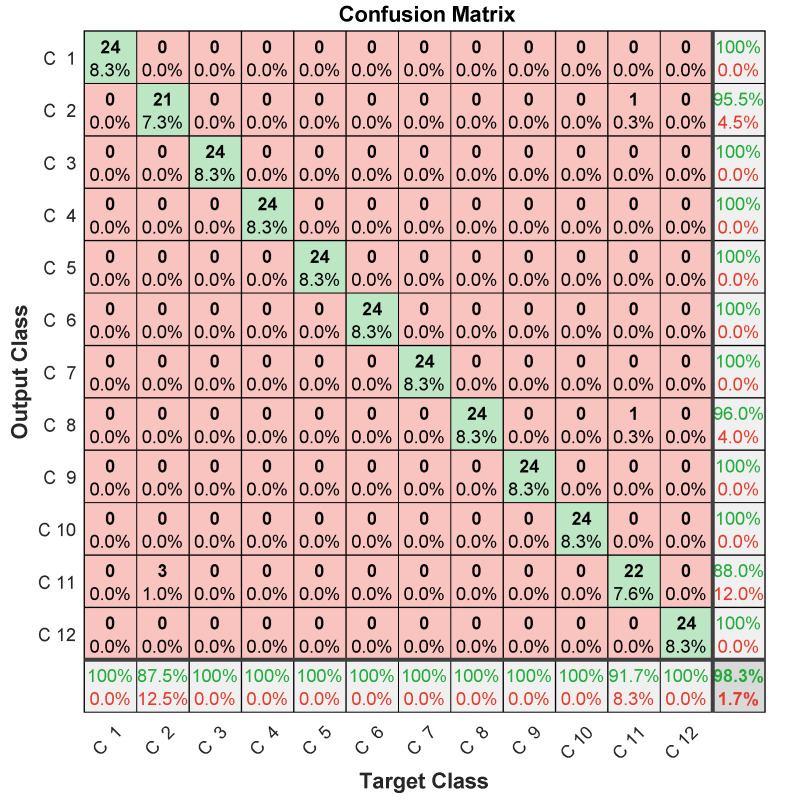
Confusion matrix (2nd microphone).

**Figure 19:**
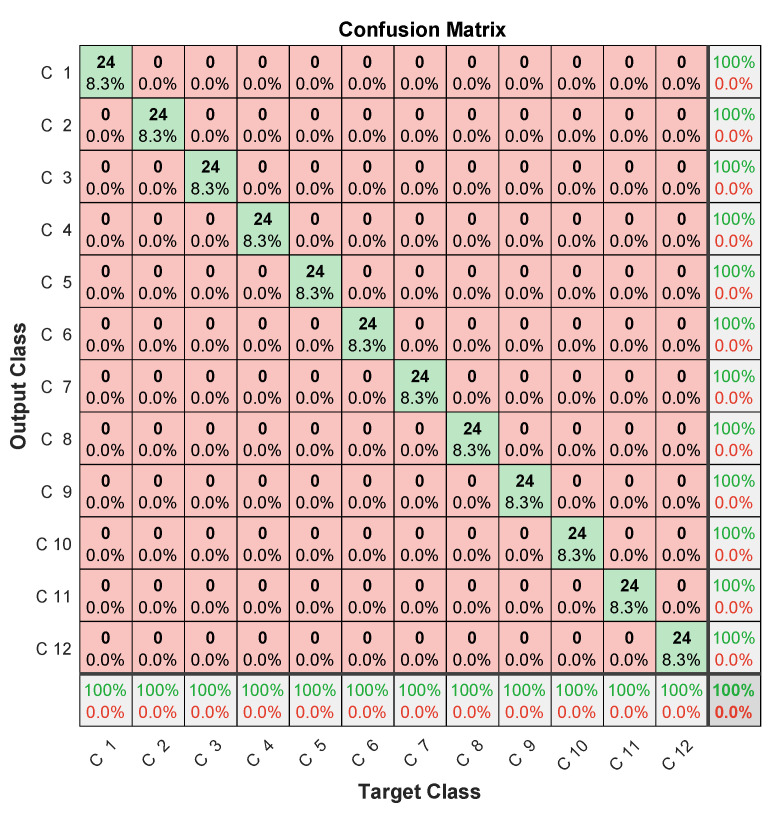
Confusion matrix (3rd microphone).

The achieved accuracy results given in Table 3 highlight the improvement brought by MFCC (about 7%) compared to that of the LPC-LSTM methodology. Confusion-matrix analysis in Figure 17, Figure 18 and Figure 19 confirmed the better classification tendency of the failure majority because of MFCC spectrum representation providing more time and frequency details from nonlinear and nonstationary signals [29], in addition to CNNs, which are known for their strong ability to extract useful features.

Despite the improvement in accuracy, this approach deals with computational-burden issues related to the convolutional layers’ slow training [42] due to successive convolutional operations during training (convolution, pooling, etc). This drawback limits convolutional networks’ usefulness for real-time diagnosis. In addition, the amount of data to be managed by a CNN is very important. It typically consists of 14×48 elements for spectral MFCC images against the 15 coefficients obtained by LPC, in addition to multiplying the number of images generated at each convolutional layer using different filters. This large amount of data can lead to memory saturation and thereby block the monitoring process, especially when monitoring several systems at the same time. Therefore, and according to confusion-matrix analysis (Figure 12, Figure 13 and Figure 14) and the disadvantages of the MFCC–CNN–LSTM approach, multisensor data fusion was adopted to improve the obtained results using the LPC–LSTM approach.

### 3.6. LPC–LSTM Early Fusion-Based Failure Diagnosis

A machine-learning literature review for classification or regression highlights penalizing a technique over another for accuracy enhancement. Analysis of other metrics such as the confusion matrix helps in improving the prediction results, with simple methods such as multichannel data fusion [20,43].

The main objective of this study was to show the effectiveness of early fusion for failure-diagnosis performance enhancement. In this context, signal merging allows for extracting discriminant features between different obtained classes from different sensors. This leads to better prediction results than those by separately using each signal. Early fusion is a machine-learning solution where fusion is ensured when training a learning network. This allows for collecting a set of features related to each class from input signals while leading to better efficiency and a higher confidence.

**Figure 20:**
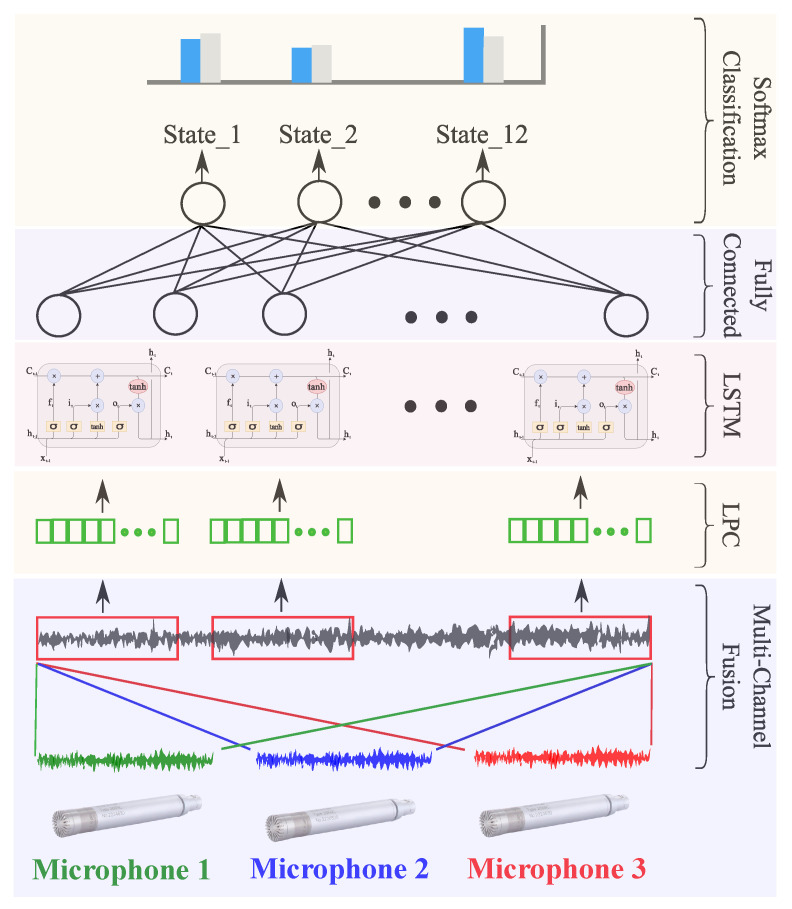
Early fusion of 3 microphones.

**Figure 21:**
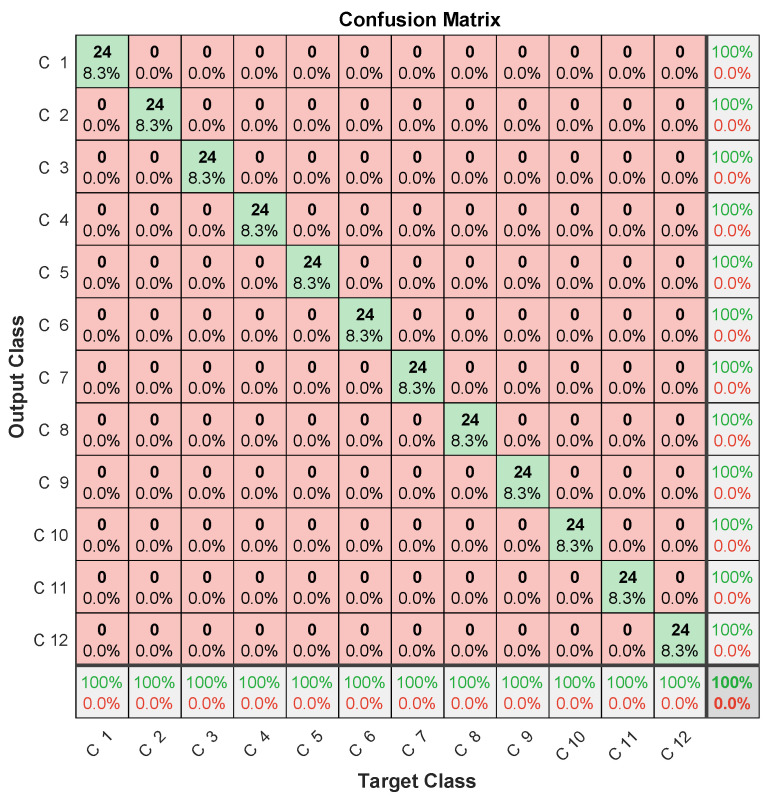
Confusion matrix (three channels’ fusion).

In this context, the three microphones’ signals are processed by LPC, as shown in Section 3.2, and the 45 obtained coefficients from the three signals (15 from each signal) are input to the learning network as shown in Figure 20. Learning then allows for the discriminating selection of the features of each class (each microphone). The obtained confusion matrix after fault diagnosis is shown in Figure 21, which clearly highlights the benefit of using early fusion, as 100% failure-diagnosis accuracy is achieved, compared to less than 90% for the same signals used separately, as shown in Table 4.

The achieved results clearly show the value of multisensor data fusion compared to that of a monosensor approach. This is mainly due to the difficulty of determining the monomicrophone optimal position to capture the maximal amount of information, especially without prior knowledge of the likely fault source. In addition, for complex machines, there may be interferences from multiple faults. These interferences influence microphones in different ways depending on the orientation and the distance from the sources of interfering faults [38,44].

The data-fusion technique based on LPC–LSTM led to encouraging results compared to those of other techniques. This is due to the small amount of postprocessing data (15 coefficients) compared to the original signal size or a transform giving a signal of significant length, such as the spectrum used for fusion in [45]. In addition, convolutional steps suffering from slow training speed [42] are not required, such as in the case of the MFCC–CNN–LSTM approach and image fusion in [46].

## 4. Conclusions

This paper provided a comparative study of two machine-learning-based approaches for gearbox failure diagnosis. The first used linear predictive coefficients for signal processing and long short-term memory for learning, while the second was based on mel-frequency cepstral coefficients for signal processing, a convolutional neural network for feature extraction, and long short-term memory for classification. In this context, the objective was to clearly highlight the importance of signal processing before learning. In addition to highlighting the advantage of using mel-frequency cepstral coefficients to enhance failure-diagnosis accuracy, there is room to further improve accuracy using multisensor data fusion. Indeed, this allows for reducing the interpretation time of each result of microphone diagnosis, in addition to improving diagnostic reliability and accuracy.

The proposed gearbox failure diagnosis methodologies were evaluated using an experimental dataset built from a specific test bench with gearbox and bearing failures.

Future investigations will focus on the optimization of learning-network hyperparameters to decrease training time and increase the number of diagnosed failures.

## Figures and Tables

**Table 1:** Simulated failure classes.

		Gear States			
		Healthy	Broken Side	Broken Tooth	Notched
Bearing states	Inner race defect	C1	C4	C7	C10
	Healthy	C2	C5	C8	C11
	Rusty	C3	C6	C9	C12

**Table 2:** LPC–LSTM methodology accuracy evaluation.

	1st Microphone	2nd Microphone	3rd Microphone
**Accuracy**	89.58%	90.28%	88.89%

**Table 3:** MFCC–CNN–LSTM methodology accuracy evaluation.

	1st Microphone	2nd Microphone	3rd Microphone
**Accuracy**	97.9%	98.3%	100%

**Table 4:** LPC–LSTM early fusion methodology accuracy evaluation.

	1st Microphone	2nd Microphone	3rd Microphone	Fusion
**Accuracy**	89.58%	90.28%	88.89%	100%

## Data Availability

Available upon request from the authors.

## References

[1] Wang T., Dong J., Xie T., Diallo D., Benbouzid M. (2019). A Self-Learning Fault Diagnosis Strategy Based on Multi-Model Fusion. Information.

[2] Li H., Huang J., Yang X., Luo J., Zhang L., Pang Y. (2020). Fault Diagnosis for Rotating Machinery Using Multiscale Permutation Entropy and Convolutional Neural Networks. Entropy.

[3] Ye X., Hu Y., Shen J., Chen C., Zhai G. (2021). An Adaptive Optimized TVF-EMD Based on a Sparsity-Impact Measure Index for Bearing Incipient Fault Diagnosis. IEEE Trans. Instrum. Meas..

[4] Khan S., Yairi T. (2018). A review on the application of deep learning in system health management. Mech. Syst. Signal Process..

[5] Benbouzid M. (2020). Signal Processing for Fault Detection and Diagnosis in Electric Machines and Systems.

[6] Khamoudj C.E., Si-Tayeb-Benbouzid F., Benatchba K., Benbouzid M., Djaafri A. (2020). A Learning Variable Neighborhood Search Approach for Induction Machines Bearing Failures Detection and Diagnosis. Energies.

[7] Tang G., Tian T. (2020). Compound Fault Diagnosis of Rolling Bearing Based on Singular Negentropy Difference Spectrum and Integrated Fast Spectral Correlation. Entropy.

[8] Delpha C., Diallo D., Harmouche J., Benbouzid M., Amirat Y., Elbouchikhi E. (2020). Bearing Fault Diagnosis in Rotating Machines. Electr. Syst. 2 Diagn. Progn..

[9] Xiao Y., Xue J., Zhang L., Wang Y., Li M. (2021). Misalignment Fault Diagnosis for Wind Turbines Based on Information Fusion. Entropy.

[10] Amirat Y., Choqueuse V., Benbouzid M. (2013). EEMD-based wind turbine bearing failure detection using the generator stator current homopolar component. Mech. Syst. Signal Process..

[11] Zhang Y., Li X., Gao L., Li P. (2018). A new subset based deep feature learning method for intelligent fault diagnosis of bearing. Expert Syst. Appl..

[12] Mao W., Sun B., Wang L. (2021). A New Deep Dual Temporal Domain Adaptation Method for Online Detection of Bearings Early Fault. Entropy.

[13] Peimankar A., Puthusserypady S. (2021). DENS-ECG: A deep learning approach for ECG signal delineation. Expert Syst. Appl..

[14] Berghout T., Mouss L.H., Bentrcia T., Elbouchikhi E., Benbouzid M. (2021). A deep supervised learning approach for condition-based maintenance of naval propulsion systems. Ocean. Eng..

[15] Xu Z., Li C., Yang Y. (2020). Fault diagnosis of rolling bearing of wind turbines based on the Variational Mode Decomposition and Deep Convolutional Neural Networks. Appl. Soft Comput..

[16] Qiao M., Yan S., Tang X., Xu C. (2020). Deep Convolutional and LSTM Recurrent Neural Networks for Rolling Bearing Fault Diagnosis Under Strong Noises and Variable Loads. IEEE Access.

[17] Wang Q., Zhu X., Ni Y., Gu L., Zhu H. (2020). Blockchain for the IoT and industrial IoT: A review. Internet Things.

[18] Lopez-Arevalo I., Aldana-Bobadilla E., Molina-Villegas A., Galeana-Zapién H., Muñiz-Sanchez V., Gausin-Valle S. (2020). A Memory-Efficient Encoding Method for Processing Mixed-Type Data on Machine Learning. Entropy.

[19] Angelopoulos A., Michailidis E.T., Nomikos N., Trakadas P., Hatziefremidis A., Voliotis S., Zahariadis T. (2019). Tackling Faults in the Industry 4.0 Era—A Survey of Machine-Learning Solutions and Key Aspects. Sensors.

[20] Ghosh N., Paul R., Maity S., Maity K., Saha S. (2020). Fault Matters: Sensor data fusion for detection of faults using Dempster–Shafer theory of evidence in IoT-based applications. Expert Syst. Appl..

[21] Cheng C., Wang J., Chen H., Chen Z., Luo H., Xie P. (2020). A Review of Intelligent Fault Diagnosis for High-Speed Trains: Qualitative Approaches. Entropy.

[22] Abdul Z.K., Al-Talabani A.K., Ramadan D.O. (2020). A Hybrid Temporal Feature for Gear Fault Diagnosis Using the Long Short Term Memory. IEEE Sens. J..

[23] Lei J., Liu C., Jiang D. (2019). Fault diagnosis of wind turbine based on Long Short-term memory networks. Renew. Energy.

[24] Yang J., Guo Y., Zhao W. (2019). Long short-term memory neural network based fault detection and isolation for electro-mechanical actuators. Neurocomputing.

[25] Hao S., Ge F.X., Li Y., Jiang J. (2020). Multisensor bearing fault diagnosis based on one-dimensional convolutional long short-term memory networks. Measurement.

[26] Park P., Marco P.D., Shin H., Bang J. (2019). Fault Detection and Diagnosis Using Combined Autoencoder and Long Short-Term Memory Network. Sensors.

[27] An Q., Tao Z., Xu X., Mansori M.E., Chen M. (2020). A data-driven model for milling tool remaining useful life prediction with convolutional and stacked LSTM network. Measurement.

[28] Dibaj A., Ettefagh M.M., Hassannejad R., Ehghaghi M.B. (2021). A hybrid fine-tuned VMD and CNN scheme for untrained compound fault diagnosis of rotating machinery with unequal-severity faults. Expert Syst. Appl..

[29] Akpudo U.E., Hur J.W. (2021). A Cost-Efficient MFCC-Based Fault Detection and Isolation Technology for Electromagnetic Pumps. Electronics.

[30] Das A., Guha S., Singh P.K., Ahmadian A., Senu N., Sarkar R. (2020). A Hybrid Meta-Heuristic Feature Selection Method for Identification of Indian Spoken Languages From Audio Signals. IEEE Access.

[31] Wu C., Jiang P., Ding C., Feng F., Chen T. (2019). Intelligent fault diagnosis of rotating machinery based on one-dimensional convolutional neural network. Comput. Ind..

[32] Xie T., Wang T., Diallo D., Razik H. (2020). Imbalance Fault Detection Based on the Integrated Analysis Strategy for Marine Current Turbines under Variable Current Speed. Entropy.

[33] Amirat Y., Benbouzid M., Wang T., Bacha K., Feld G. (2018). EEMD-based notch filter for induction machine bearing faults detection. Appl. Acoust..

[34] Bäckström T. (2017). Speech Coding: With Code-Excited Linear Prediction.

[35] Ebrahimnezhad H., Khoshnoud S. (2013). Classification of arrhythmias using linear predictive coefficients and probabilistic neural network. Appl. Med. Inform..

[36] Li X., Zhang W., Ding Q. (2019). Deep learning-based remaining useful life estimation of bearings using multi-scale feature extraction. Reliab. Eng. Syst. Saf..

[37] Guoping Z. (2019). On the confusion matrix in credit scoring and its analytical properties. Commun. Stat. Theory Methods.

[38] Wang R., Liu F., Hou F., Jiang W., Hou Q., Yu L. (2020). A Non-Contact Fault Diagnosis Method for Rolling Bearings Based on Acoustic Imaging and Convolutional Neural Networks. IEEE Access.

[39] Wang X., Mao D., Li X. (2021). Bearing fault diagnosis based on vibro-acoustic data fusion and 1D-CNN network. Measurement.

[40] Zhang D., Stewart E., Entezami M., Roberts C., Yu D. (2020). Intelligent acoustic-based fault diagnosis of roller bearings using a deep graph convolutional network. Measurement.

[41] Glowacz A., Tadeusiewicz R., Legutko S., Caesarendra W., Irfan M., Liu H., Brumercik F., Gutten M., Sulowicz M., Daviu J.A.A. (2021). Fault diagnosis of angle grinders and electric impact drills using acoustic signals. Appl. Acoust..

[42] Wang W., Yang J., Chen M., Wang P. (2019). A Light CNN for End-to-End Car License Plates Detection and Recognition. IEEE Access.

[43] Liu J., Li T., Xie P., Du S., Teng F., Yang X. (2020). Urban big data fusion based on deep learning: An overview. Inf. Fusion.

[44] Vamsi I., Sabareesh G., Penumakala P. (2019). Comparison of condition monitoring techniques in assessing fault severity for a wind turbine gearbox under non-stationary loading. Mech. Syst. Signal Process..

[45] Gogate M., Dashtipour K., Hussain A. (2020). Visual Speech In Real Noisy Environments (VISION): A Novel Benchmark Dataset and Deep Learning-Based Baseline System. Proc. Interspeech.

[46] Zhang L., Gao H., Wen J., Li S., Liu Q. (2017). A deep learning-based recognition method for degradation monitoring of ball screw with multi-sensor data fusion. Microelectron. Reliab..

